# Acquired resistance L747S mutation in an epidermal growth factor receptor-tyrosine kinase inhibitor-naïve patient: A report of three cases

**DOI:** 10.3892/ol.2013.1705

**Published:** 2013-11-25

**Authors:** FUMIHIRO YAMAGUCHI, KUNIHIKO FUKUCHI, YOHEI YAMAZAKI, HIROMI TAKAYASU, SAKIKO TAZAWA, HIDETSUGU TATENO, EISUKE KATO, AYA WAKABAYASHI, MAMI FUJIMORI, TAKUYA IWASAKI, MAKOTO HAYASHI, YUTAKA TSUCHIYA, JUN YAMASHITA, NORIKAZU TAKEDA, FUMIO KOKUBU

**Affiliations:** 1 Department of Respiratory Medicine, Showa University Fujigaoka Hospital, Aoba-ku, Yokohama 227-8501, Japan; 2 Department of Clinical Pathology, Showa University School of Medicine, Shinagawa-ku, Tokyo 142-8666, Japan

**Keywords:** L747S, epidermal growth factor receptor-tyrosine kinase inhibitor resistance mutation, small cell lung carcinoma

## Abstract

The purpose of the present study was to report cases of epidermal growth factor receptor-tyrosine kinase inhibitor (EGFR-TKI)-naïve patients carrying a mutation associated with acquired resistance to the drug. Gene alterations in 77 lung carcinoma patients were analyzed by collecting and studying curette lavage fluid at the time of diagnosis. PCRs were performed to amplify mutation hotspot regions in *EGFR* genes. The PCR products were direct-sequenced and the mutations confirmed by resequencing using different primers. Case 1 was a 78-year-old Japanese male diagnosed with stage IB lung adenocarcinoma who was found to have two *EGFR* mutations, G719S and L747S. Case 2 was a 73-year-old Japanese male diagnosed with stage IV squamous cell lung carcinoma and bone metastasis who had the *EGFR* mutation, L747S. Case 3 was an 82-year-old Japanese male diagnosed with hyponatremia due to inappropriate secretion of antidiuretic hormone and stage IIIB small cell lung carcinoma (SCLC) who had the *EGFR* mutation, L747S. Thus, the *EGFR* mutation L747S associated with acquired EGFR-TKI resistance was detected in two non-small cell lung carcinoma (NSCLC) patients and one SCLC patient, none of whom had ever received EGFR-TKI. The patients were current smokers with stages at diagnosis ranging from IB to IV, and their initial tumors contained resistant clones carrying L747S. L747S may be associated with primary resistance. To the best of our knowledge, this study is the first report of an *EGFR* mutation associated with resistance to EGFR-TKI in SCLC patients. The early detection of EGFR-TKI resistance mutations may be beneficial in making treatment decisions for lung carcinoma patients, including those with SCLC.

## Introduction

The efficacy of epidermal growth factor receptor-tyrosine kinase inhibitor (EGFR-TKI) for treatment of non-small cell lung carcinoma (NSCLC) patients with *EGFR* activating mutations is well established. Several prospective studies have observed that first-line EGFR-TKI treatment leads to longer progression-free survival in NSCLC patients with such *EGFR* mutations compared with platinum-based doublet chemotherapy (1–4). Hence the presence of *EGFR-*activating mutations can be used to determine whether to administer EGFR-TKI to NSCLC patients. In addition, *EGFR* mutations associated with primary resistance or acquired resistance to EGFR-TKI have been identified (5). Therefore, the ability to detect both types of *EGFR* mutations is important in making treatment decisions for NSCLC patients. To date, there have been numerous reports of *EGFR* mutations, including alterations associated with drug sensitivity or drug resistance. *EGFR-*activating mutations, specifically a deletion in exon 19 and the missense mutation L858R in exon 21, have been reported most frequently, accounting for >90% of mutations in *EGFR*(6,7). By contrast, insertions in exon 20 and missense mutation T790M in exon 21 are described as primary resistance mutations for EGFR-TKI. Four *EGFR* mutations have been associated with acquired resistance to EGFR-TKI, L747S, D761Y, T790M and T854A (8,9), but the mechanism for acquiring these mutations remains unclear. In the present study, L747S was identified in three patients who had never received EGFR-TKI therapy, indicating that the initial tumor contained resistant clones carrying L747S in varying proportions.

## Case report

### Method

Gene alterations in 77 lung carcinoma patients were analyzed by collecting and studying curette lavage fluid at the time of diagnosis. DNA was extracted from cells attached to the curette and PCR was performed to amplify mutation hotspot regions in the *EGFR* genes, as described previously (10). The PCR products were direct-sequenced and the mutations were then confirmed by resequencing using different primers. Approval for the study was obtained in advance from the Ethics Committee for Genomic Research at Showa University (Tokyo, Japan; approval number 113). The patients provided written informed consent.

### Results

Overall, 27% (21 of 77) were found with *EGFR* mutations, including L747S detected in three patients.

#### Case 1

Table I shows the patient characteristics and their mutation statuses. A 78-year-old Japanese male with a current smoking history of 45 pack-years was referred to Showa University Fujiguoka Hospital (Yokohama, Japan) due to a screen-detected abnormal chest X-ray. As a result, the patient was diagnosed with stage IB lung adenocarcinoma. A computed-tomography (CT) scan of the chest at diagnosis revealed a primary tumor, 15-mm in size, in the right upper lobe. The levels of the tumor markers, carcinoembryonic antigen (CEA), CYFRA21-1 and pro-gastrin-releasing peptide (Pro-GRP), were within normal limits. The lobe was completely resected with a lymph node dissection. No recurrence was observed during two years after surgery. The tumor was found to have two *EGFR* mutations, G719S and L747S. Neither mutation was detected in the genomic DNA extracted from the normal tissue of the lung.

#### Case 2

A 73-year-old Japanese male with a smoking history of 80 pack-years was diagnosed with stage IV squamous cell lung carcinoma and bone metastasis, found due to a chest X-ray abnormality that was detected during a routine checkup. A CT scan of the chest showed that the majority of the right upper lobe consisted of a neoplasm, and that the tumor had infiltrated the mediastinum. The levels of the tumor markers, CEA, CYFRA21-1 and Pro-GRP, were within normal limits. The patient possessed the *EGFR* mutation, L747S. Single-agent chemotherapy consisting of 1000 mg/m^2^ gemcitabine was administered at on days 1, 8 and 15. The therapy was repeated every four weeks for two cycles; however, the patient failed this therapy and succumbed within four months of diagnosis. Neither normal tissue nor whole blood was available for further analysis.

#### Case 3

An 82-year-old Japanese male with a smoking history of 30 pack-years was hospitalized (Showa University Fujiguoka Hospital) due to extreme fatigue. The patient’s serum sodium level was 113 mEq/ml upon admission. The patient was diagnosed with hyponatremia, due to the inappropriate secretion of antidiuretic hormone, and with stage IIIB small cell lung carcinoma (SCLC). As shown in Table I, the tumor markers, CEA and Pro-GRP, were elevated with values of 10.5 and 468.1 pg/ml, respectively. The patient possessed the *EGFR* mutation, L747S. The patient received carboplatin at a dose of AUC 5.0 every four weeks on day 1, and etoposide at a dose of 80 mg/m^2^ every four weeks on days 1, 2 and 3. A total of 16 cycles were administered in the last two years. L747S was not detected in the genomic DNA from a whole blood sample.

#### Sequencing

**Figure 1:**
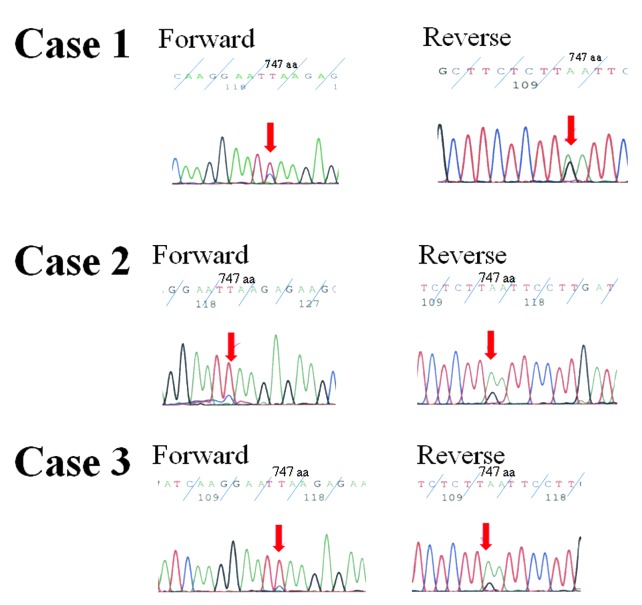
Identification of L747S in three lung carcinoma patients. Sequencing of *EGFR* exon 19 demonstrates a T to C (reverse A to G) base-pair change (arrows) at amino acid 747. Samples obtained in three lung carcinoma patients demonstrated the appearance of this mutation. *EGFR*, epidermal growth factor receptor.

As shown in Fig. 1, samples from each subject were direct-sequenced and the mutations were then confirmed by sequencing using different forward and reverse primers. In summary, the present study revealed that the *EGFR* mutation, L747S, was detected in male smokers with >30 pack-years each, and who were diagnosed with adenocarcinoma, squamous cell carcinoma and SCLC. Among these cases, two of the L747S mutations were confirmed to be somatic.

## Discussion

The purpose of this study was to report cases of EGFR-TKI-naïve patients carrying the *EGFR* mutation, L747S, which is associated with acquired resistance to the drug. Previous studies have revealed that acquired resistance mutations, including L747S, are rare (8,9). There have also been few reports of L747S detection in lung carcinoma patients who have not yet received treatment (11,12). In the present study, 3.9% (3/77) EGFR-TKI-naïve patients were identified with L747S, as reported previously (10). Tumor cells carrying L747S may have an advantage in carcinoma progression. L747S was identified in two NSCLC patients and in one SCLC patient, all of whom were male smokers. L747S was not associated with staging. As summarized in Table I, no patients showed overlap with a deletion in exon 19 or L858R in exon 21, whereas one adenocarcinoma patient carried G719S in exon 18. No pathogenesis of an acquired resistance mutation was evident. Two possible mechanisms of acquiring EGFR-TKI resistance have been proposed. One hypothesis is that the initial tumor includes cells carrying resistance mutations that exist prior to EGFR-TKI therapy. The second hypothesis is that tumor cells acquire novel resistance mutations during therapy. These two hypotheses assume that resistant clones are selected through EGFR-TKI therapy. The present study revealed that three smokers who had never received EGFR-TKI therapy harbored the *EGFR* mutation, L747S. The initial tumor in each case likely contained resistant clones carrying L747S in varying proportions, consistent with the former hypothesis. This mutation may also be associated with the primary resistance to EGFR-TKI therapy. The presence of L747S in *EGFR* should therefore be noted, particularly for patients with *EGFR*-activating mutations, since dose escalation of EGFR-TKI may overcome resistance due to the mutation (13,14). Notably, L747S may be detected only by direct-sequencing, and not by the Scorpion-amplification refractory mutation system or peptide nucleic acid-locked nucleic acid PCR clamp methods commonly used in clinical practice (15).

There have been several reports of *EGFR* mutations in SCLC (16–18), thus the mutation status of *EGFR* should be analyzed in NSCLC and SCLC. EGFR-TKI has been shown to induce a partial response in SCLC patients carrying *EGFR*-activating mutations, which are described as SCLC combined adenocarcinoma components (19–21). In case 3, the Pro-GRP and CEA levels were elevated simultaneously, indicating that adenocarcinoma cells were included in the tumor. Studies have revealed a histological transformation from NSCLC into SCLC in combination with alterations of tumor markers in *EGFR* mutant patients who acquired EGFR-TKI resistance (22,23). The present study is the first report showing an *EGFR* mutation associated with resistance to EGFR-TKI therapy in an SCLC patient. This indicates that the mutation status in SCLC requires further investigation, and that it will be useful to detect *EGFR*-activating mutations or resistance mutations of EGFR-TKI in SCLC and NSCLC patients.

The *EGFR* mutation, L747S, was detected in two NSCLC patients and one SCLC patient, none of whom had ever received EGFR-TKI therapy. In addition to clonal selection of resistant cells in the initial tumor, cells carrying L747S may predominate following EGFR-TKI therapy. L747S may be associated with primary EGFR-TKI resistance. The presence of L747S in *EGFR* should therefore be considered, particularly for patients with *EGFR*-activating mutations. The early detection of EGFR-TKI resistance mutations may be beneficial in making treatment decisions for lung carcinoma patients. In the future, analyses of *EGFR* mutations in lung carcinoma, including SCLC, should be continued.

## Figures and Tables

**Table I:** Clinical characteristics and mutation status.

Characteristic	Case 1	Case 2	Case 3
Age, years	78	73	82
Gender	M	M	M
Stage	IB	IV	IIIB
Cytological diagnosis	Ad	Sq	SCLC
Tobacco, pack-years	45	80	30
*EGFR* mutation	G719S, L747S	L747S	L747S
Tumor marker			
CEA, ng/ml	1.7	1.9	10.5
CYFRA21-1, ng/ml	2.0	2.4	1.4
Pro-GRP, pg/ml	25.3	27.2	468.1

M, Male; Ad, adenocarcinoma; Sq, sequamous cell carcinoma; SCLC, small cell lung carcinoma; *EGFR*, epidermal growth factor receptor; CEA, carcinoembryonic antigen; Pro-GRP, pro-gastrin-releasing peptide.
